# Abstract of the Proceedings of the Buffalo Medical Association

**Published:** 1856-11

**Authors:** Sanford B. Hunt


ART. II.—*Abstract of the Proceedings of the Buffalo Medical Association.*

TUESDAY EVENING, Oct. 7, 1856.

The association met.

Present—The President, Dr. Eastman, in the chair. Drs. Hamilton, Lewis, Treat, Newman, Wilcox, Lemon, Rogers, White, Almy, Dayton, Perkins, Strong, Hunt, Gould, Mixer, Miner, Hawley, Baker, and Rochester.

The minutes of the preceding meeting were read and approved.

Dr. L. G. Hamm, of Williamsville, was chosen an honorary member, on motion of Dr. Wilcox.

Dr. Wm. Van Pelt, of Williamsville, was chosen an honorary member, on motion of Dr. Hamilton.

Dr. J. M. Perkins was proposed for membership by Dr. White.

*Prof. Hamilton* then exhibited a specimen of diseased bone, accompanied by the following description:

*A specimen of Chronic Abscess of the Bone, occurring in the lower end of the Tibia.* In 1844 the leg was broken about three inches above the ankle joint; the fracture united kindly, but from that day until this, he has suffered more or less pain in the limb. At length an opening occurred in the lower part of the leg, and pus was discharging. Dr. Hamilton amputated the leg near its middle, on the 4th of last month.

The inflammation and consequent softening of the bone extends to about six inches above the joint, the whole of this portion being enlarged by an expansion of its structure, and by the conversion of its lamellated tissue into cellular, so that the structure of that portion of the diaphysis which is involved can not be distinguished from the epiphysis.

The cavity of the abscess is in the lower end of the bone, and is two inches in length in its long diameter, and one in its short. At two points the walls have been perforated by ulceration or absorption, forming cloacæ. The whole was lined with a perfectly organized and quite firm pyogenic membrane. The fibula has not partaken in the disease.

It was believed that amputation would afford the only remedy, as the disease had existed so long a time, and the openings in the bone, which were free and had existed also a long time, had failed to give any relief.

*Prof. Hamilton* also exhibited a specimen of osteo-sarcoma, with the following account:

*A specimen of Osteo-Sarcoma.* On the first day of January, 1854, Harriet Young, the girl from whom this specimen was obtained, at that time 20 years old, fell upon the frozen ground, striking upon her right hip, since which time she has had a pain in the right thigh, and the disease has gradually progressed. At length, on the 2d of this month, she died, worn out with pain. Dr. Hamilton had seen her and counselled her several times, but was not present at the autopsy. Dr. Storck, however, an intelligent German physician, and a member of this society, procured for him this specimen, and gave him this brief account of the case.

The thigh measured in diameter 21 inches, and as its diameters were nearly equal it probably measured over five feet in circumference. The enlargement extended from just below the head of the bone to a point two or three inches above the lower end, involving only the diaphysis, and not at all affecting the epiphysis. The tibia was diseased in the same manner, but to a less extent. In the case of this latter bone, also, the epiphysis was not involved, so that the knee joint, as well as the hip joint, were unaffected. Nearly all of the bones of the pelvis on this side were implicated.

The cells of the diseased femur were filled with a yellowish serum; and the whole mass, through its entire thickness, was of the same structure and appearance with this specimen, which is only a portion of its outer shell.

The form of the mass is pretty uniformly ovoid, the surface being only slightly broken by the canals and cells which open it every where. It is covered in portions by a very much overgrown periosteum, lying in bands irregularly spread out, with a coarse, skinny, fibrous structure. In portions this structure is replaced with, or it has degenerated into small tuft-like masses, which appear soft and velvety to the naked eye, but under a magnifying glass are seen to be porous, like a honeycomb. These masses vary from one line to several lines in diameter, and are one or two lines in thickness. Underneath this periosteum, &c., is a thin bony crust. The interior is filled up with cells and irregular rays or lines of bone which in many parts become quite compact, and in others expand into cells. None of the cells in this specimen exceed three quarters of an inch in diameter; and they are all lined with a thin, shining, pink colored, and somewhat reticulated membrane.

Its structure is throughout bony; the bone resting in a fibrous stroma, and it is difficult to say which element preponderates.

*Prof. Hamilton* next called the attention of the association to a specimen of multilocular cyst:

I wish to present to the society a specimen of *congenital, multilocular cyst*, which is also *sero-sanguineous* in the character of its contents.

The little girl from whom this specimen was obtained, Louisa McCaa, is eleven years old. At birth a small tumor was noticed on the left cheek near the angle of the jaw, a portion of which was firm, and a portion elastic, as if containing fluid. Dr. Van Aernam, of Chatauque Co., had for some time supposed it to be connected with the parotid gland.

In May of this year, the child was brought to me. The tumor was then so large as to cover nearly the whole of the left cheek. It was elastic. With an exploring needle, I ascertained that it contained a bloody serum, and that it was multilocular.

A violent inflammation and great swelling followed this small wound, which only subsided after a fortnight, and a bloody serum continued to discharge many days.

On the 24th of June I gave her chloroform and proceeded to extirpate the tumor, assisted by Dr. Boardman, and Messrs. Mason, Flint, &c. I soon ascertained that the mass was composed of the parotid gland; the portio dura and the external carotid artery passing through it. The first was cut, and also the trunk of the temporal artery. I removed, with great labor, all of the tumor lying upon the cheek, and descended as far as was practicable into the space behind the jaw.

The tumor itself consisted of an innumerable aggregation of cysts or of spaces, varying in size from a pin's head to the size of a pullet's egg: the largest cysts being those which were nearest the surface, those which lay most profound being scarcely discernable as cysts, and finally the cysts seemed to be entirely lost in the natural tissue of the gland.

The walls of these cysts were in the main thin and smooth, but inlaid with duplications or columns resembling very much the columnæ carniæ and cordæ tendinæ of the heart.

The contents were uniformly the same, serum colored with blood, and as the walls were diaphanous, the smaller cysts looked like dilated veins.

On every side the cysts were firmly adherent to the adjacent tissues, and the vascularity of these tissues was very remarkable. We tied a large number of arteries.

I did not close the wound until all bleeding had ceased, and the dressings then applied were very light and simple.

Within a few hours after the operation was completed, a swelling commenced around the margins of the cavity, which soon became very tense and painful. A bloody serum also continued to ooze for several days.

This interesting little patient, whose courage was always equal to her suffering, remained under my charge several weeks and then returned home. I have since learned, indirectly, that her recovery was complete, but with how much disfigurement of the face I am unable to say.

This is the second time I have met with a tumor of this character. I mean a multilocular cystic tumor.

In the first instance referred to, the child, a fine healthy boy, was one year old, of light complexion and sandy hair. At birth a small purplish spot, like an erectile narous, in which the venous character predominated, was seen on the left shoulder blade. At the time of my operation, Feb. 23, 1855, it was of the size of a cocoa nut, elastic, smooth, and without tenderness.

I opened it freely with a simple incision, but did not attempt to dissect it out. A number of cysts came consecutively into sight and were opened. The cysts contained a thin, yellow serum. There was not at this time any hæmorrhage of consequence.

Twenty-four hours after the operation I found the sac filled with blood. I first removed the clot and seared the wound with a hot iron, but as the bleeding did not cease, I opened the sac and tied numerous small vessels in the partitions of the various cysts.

In twenty-four hours it was again full of blood, and at the end of forty-eight hours I emptied it thoroughly, and opened nine more cysts which I had not seen before, but which were now very large. I again seared all the sacs with a hot iron, and still farther sought to control the bleeding with perchlor ferri. The bleeding did not return, but the child died, partly from the shock, and partly from the loss of blood, on the fourth day after the operation.

I have seen one other case of encysted tumor, whose walls were exceedingly vascular, and from which a troublesome hæmorrhage has followed. This was the case of a young miss ten years old, with a simple encysted tumor of three years' growth, situated on the back, over the lower angle of the scapula. The hæmorrhage in this example continued four days, and was finally arrested, with great difficulty, by the actual cautery. I have seen, also, other cystic tumors containing a sero-sanguineous fluid, one of which at least was congenital.

It is to the multilocular cystic tumors, however, that I wish especially to call your attention, and of which this specimen is an example. Since they are fortunately very rare, and I have reason to believe, there character is not so simple as they at first seem to be. There is, in fact, a strong probability that they are all more or less erectile.

The example before you corresponds to the usual history of erectile tumors in the following circumstances: It was congenital. It was painless in its growth. Its walls and partitions were exceedingly vascular, and this vascularity extended much beyond its outer walls, and after excision, the neighboring tissues continued not only to bleed but became rapidly and intensely swollen, as if from vascular erythema and congestion.

In its minute anatomy, also, it bears a striking analogy to erectile tumors not only, but to erectile and cavernous tissue in its normal state; the smaller spaces, or the cells in those portions where the disease had not so fully developed itself, resembling the cells of many purely erectile tumors, and especially the cells of the corpora cavernosa of the penis. The contents also of the cells was a bloody serum.

Similar multilocular cysts have been mentioned by pathologists. Mr. Paget records two, in both of which the septa were fasciculated like the walls of the right auricle, which were removed successfully. Mr. Paget thinks both of them may have had their origin in vascular nævi. That such was the fact with my little patient, who was only a year old at the time of the operation, the early history clearly shows. This specimen which I show now seems to have been a development of the natural structure of the parotid gland, but it was still erectile and congenital, and may properly be called a nævus.

It was impossible to remove this tumor entire without previous ligature of the carotid artery, and I chose to remove as much as possible and trust to suppuration.

I am not yet prepared to say how I should proceed in another similar case. It is certainly very difficult to cut them out entire, owing to the vascularity of all the structures adjacent, and to the intimate union which their walls have every where with these structures. In some regions such dissections would be totally impracticable. I should be unwilling again to trust to simple incision, or to partial exsection on account both of the danger of hæmorrhage and of the probable inefficiency of these measures. In the examples quoted by Paget, one of which was on the back and one on the pubis, and neither of which were congenital, the tumors were safely exsected; but the patients were much older than either of mine, and the writer has not sufficiently described the embarrassments of the operation, if any existed, to enable me to judge of its applicability to other cases.

I presume for the present we shall only attempt to cut them out entire, but my own experience might warn surgeons not to regard such procedures as always unattended with serious difficulties.

*Prof. Hamilton* also exhibited a very large ovarian tumor, successfully removed from a lady, by Dr. Nelson Winton, of Havana, N. Y., a description of which will be found elsewhere in this Journal. Dr. H. says:

"I present you also with a specimen of an encysted ovarian tumor, successfully removed the first of Sept. last, from a lady in Havana, N. Y., æt. 35, by Nelson Winton, M. D. It weighed at the time of its removal, 33lbs. The patient has nearly recovered. But as Dr. Winton has consented to furnish an account of the case to the Buffalo Medical Journal, I shall not speak of it more particularly."

*Prof. Hamilton* gave the following formula for a fluid for cleansing diseased bone, for pathological purposes. It produces a beautiful white and cleanly appearance:

℞ Chloride of lime, 1 ℔.
Bi-carb. potash, 1 oz.
Water, 2 gallons.

*Prof. White,* in allusion to operations for ovariotomy, said that that operation, though severely censured by many of the leading writers in the profession, was still an open question. For himself, he was no longer a surgeon, and could therefore speak dispassionately. In estimating the eligibility of the operation, we should take into consideration the great fatality of the disease itself. Dr. Robert Lee, to gratify a splenetic feeling against a brother surgeon, had collected statistics, placing the operation in as unfavorable a light as possible. Including all Dr. Lee's cases, we find that, although in many of them erroneous diagnosis had been made, the proportion of successful cases was about one-half. Now as all these cases must have resulted fatally, if left to themselves, he regarded it as an eligible operation, even on the basis of Lee's statistics. If only forty per cent. recovered, it should be recollected that the fatality would have been 100 per cent. without the operation. Dr. Peaslee's recent operation, and Dr. Kimball's extirpation of the entire uterus, prove that large incisions may be made into the cavity of the peritoneum, under favorable circumstances, with a fair degree of propriety. Taking these facts into consideration, we should sustain surgeons who perform these formidable operations, in the hope that their present safety may be increased.

*Prof. White* then exhibited some instruments, recently brought into use for the purposes of uterine treatment. One of these was designed for dilating the *os uteri.* Dr. Mackintosh, some time ago, had proposed to effect this purpose by sponge and elm bark tents. The instrument exhibited was not unlike Simpson's sound. The distal or uterine end was divided, and on being introduced into the os uteri, the anterior portion can be pushed forward, by means of a screw in the handle. Prof. White had added a scale by which the precise degree of dilatation can be measured.

Bearing on the same subject, that of painful menstruation, was an apparatus for producing local anæsthesia in the uterus. Ever since chloroform was introduced, it has been used locally for similar purposes. Heretofore Prof. W. has been in the habit of using chloroform by means of a glass tube packed with lint saturated with chloroform, introduced into the vaginal canal, and had found this very advantageous. More recently carbonic acid gas had been used as a local anæsthetic. During the past summer Prof. W. had treated three cases of uterine cancer, in which anodynes had been worn out. In these cases he had derived much benefit from these local applications. He then exhibited an ingenious apparatus for applying either the vapor of chloroform, or carbonic acid gas, directly to the os uteri. It consisted of a generator, to which an elastic tube was attached, with a glass tube at the end, by which the gas is locally applied.

A more simple apparatus was a junk-bottle, with a tube attached.

In cases of dysmenorrhœa, he had found these applications, particularly of chloroform, very useful. Very frequently, a simple application seemed to bring on the menstrual flow, and thus entirely relieve the difficulty for that time.

*Prof. Hamilton* said it was worth while to inquire how much stimulation had to do with local anæsthesia produced by chloroform. Was the effect a directly sedative one, or was it the reäction after momentary stimulation? It is unquestionably true, that a momentary exaltation of local sensibility may be followed by anæsthesia. It may be that chloroform acts much like a vesicant, that it has a secondary sedative effect, which, however, depended very much on a primary stimulation. No experiments have been made with any sufficient care to establish the truth.

*Dr. Miner* remarked that he was surprised that the vapor of chloroform, applied in such small quantity as it must necessarily be by this tube, should produce any marked anæsthetic effect. He had no personal experience in local anæsthesia, but from his knowledge of the large amount of chloroform sometimes necessary, in order to produce general insensibility by inhalation, he should suppose that the remedy proposed would be insufficient.

*Dr. Treat* said that he could see how even the stimulating effect of chloroform might relieve dysmenorrhœ, by exciting the menstrual flow, in the same manner as ammonia. Or it might be a direct sedative, like hydrocyanic acid, which had no stimulating effect when applied directly to the eye.

*Prof. Hunt* differed. He had used the vapor of hydrocyanic acid, locally, upon the eye, very largely. Its immediate effect was always to promote a flow of blood and engorge the vessels, though its permanent operation seemed to shrivel them up and finally cut them off.

*Prof. White* was not disposed to discuss the rationale of local anæsthesia, inasmuch as he could not claim to know how much stimulation had to do with the effect produced. Fluid chloroform was certainly a stimulant. It would vesicate. How much of this power was possessed by the vapor he could not say. In reply to Dr. Miner, he would remark that the quantity of vapor was not so small as he might suppose. He should have mentioned that the generation of vapor was increased by wrapping hot cloths around the generator. The tube should also be traversed about from place to place in the vagina. The vapor is thus carried directly to the seat of pain, and whatever theoretical objections might be raised, he could assure members that anæsthesia was produced.

*Prof. Hunt* spoke of the anæsthetic effect of carbonic acid gas. Some years ago, he had known toothache cured, instantaneously, by holding a little vinegar and carbonate of soda in the mouth, in a state of effervescence. It was a domestic remedy, which he had repeatedly tried successfully on himself, but the necessity for its use no longer existing, he had allowed it to fall into disuse, until the subject was recalled by Dr. Simpson's paper.

*Prof. Hamilton* wished to suggest that there might be cases of ulceration, or inflammation of the uterus, in which the stimulating property of chloroform might do serious harm.

*Prof. White* said that no doubt that injury might thus be done, that the application of the remedy should be confined mostly to neuralgic or rheumatic cases, and it would be necessary to discriminate in cases.

*Prof. Rochester* said that, having just entered the room, he had only gathered that the discussion related to local applications to the uterus. He would state that he had once been induced to apply ætherial solution of iodine to the os uteri, in a case of induration, with a view to its absorbent action. It proved to be a very mischievous application, and, as it had been recommended, he thought it worth while to caution members against it.

*Prof. White* replied that the uterus was very patient under the action of eschartics, even of the severest character, but that for some reason it would not endure iodine.

Prof. W. then gave an account of a case of a young gentleman, who was taken ill with a swelling over the lower part of the abdomen, above the injured region. This was leeched, but continued to spread until it passed entirely around the body, and up toward the arm-pits, and down upon the thigh. The swelling was red, painful and phlegmonous, not erysipelatous, in its appearance. It crepitated under the finger and soon suppurated. It was opened in many plases, discharging much gas, and a very fœtid pus of a fœcal color. The tumor diminished, and under the use of tonics the patient seemed to be improving, when peritonitis set in and he died. The process of the swelling and suppuration, seemed to have almost entirely dissected the integument from the fascia beneath.

Prof. W. supposed that the cause of the difficulty was a local, circumscribed peritonitis, with perforation of the intestine. In no other way could he account for the presence and peculiar odor of the gas. As the patient had only died that day, he had made no *post-mortem.*

*Prof. Hamilton* detailed a case of inguinal hernia, which had never been strangulated, but which, nevertheless, suppurated with perforation of the intestine, and resulted in a swelling similar to that described by Dr. White, though not so extensive. The gas discharged was of a fœcal odor, and the case terminated in artificial anus.

On the usual inquiry as to prevalent diseases, it was found that no epidemic existed, and that the city was in a remarkably good state of health.

It was suggested that as the first Tuesday in November fell upon election day, and the meeting on that evening might interfere with the patriotism of members, the next meeting should be held on Thursday, Nov. 7.

And the association accordingly adjourned to the evening of Thursday, Nov. 7th.

SANFORD B. HUNT, Secretary.

